# Performance of 1-year-old camel calves fed a basal ration of alfalfa hay supplemented with different levels of concentrate

**DOI:** 10.1186/s12917-025-04485-6

**Published:** 2025-01-27

**Authors:** Ahmed R. Askar, Rawia Darwesh, Sabbah Allam, Galal M. Abdul Aziz, Afaf A. El Shereef, Ehab Y. Eid, Hamedi M. Kandil, Samir S. Abou El Ezz, Mohsen M. Shoukry

**Affiliations:** 1 Animal and Poultry Nutrition Department, Desert Research Center, Cairo, Egypt; 2 Animal Production Department, Faculty of Agriculture, Cairo University, Giza, Egypt; 3 Animal and Poultry Physiology Department, Desert Research Center, Cairo, Egypt; 4 Animal Production Department, National Research Centre, Cairo, Egypt

**Keywords:** Camels, Concentrate, Growth performance, Digestibility, Rumen, And blood metabolites

## Abstract

**Background:**

The use of a high-concentrate diet in fattening camels may have significant effects on growth performance and digestion as well as economic returns. This experiment was designed to study the effects of feeding different levels of concentrate in their diet on growth performance and digestion in a desert climate.

**Methods:**

Eighteen 12-month-old male camel calves were used, and divided into three treatments of six each. The concentrate was administered based on their body weight (BW) at 0.7 (low), 1.0 (medium), and 1.3% (high), with free access to alfalfa and water. The experiment lasted for 6 months, in which digestibility trials took place at 14, 16, and 18 months of age, which corresponded to 2, 4, and 6 months of the experimental period.

**Results:**

No significant variations were observed in final BW, BW changes, or average daily gain among feeding treatments. Increasing the concentrate level had a negative effect on roughage intake, impacting the roughage-to-concentrate ratio (*P* < 0.05) and neutral detergent fiber digestibility (*P* < 0.05). Increasing concentrate levels significantly increased total intake (*P* < 0.05), leading to a worse feed conversion ratio (*P* < 0.05). However, animal age had no negative effect on nutrient digestibility, and there were no interactions between concentrate supplement level and animal age. Significant increases in plasma total protein (*P* < 0.05) and urea (*P* < 0.05) were observed when the leve lof concentrate was increased. A similar trend was observed in rumen ammonia concentration. Camel calves fed low vs. medium or high levels of concentrate showed a greater rumen pH (*P* < 0.05), which was linked to a lower concentration of volatile fatty acids (*P* < 0.010).

**Conclusions:**

The present study concluded that yearling camel calves receiving different levels of concentrate with ad lib alfalfa hay could cover their nutrient requirements for maintenance and growth with a daily gain of 630 g/day when the level of concentrate was limited to 0.7% of BW and the total intake was only around 1.65% of BW, or 70.6 g/kg metabolic BW.

## Introduction

Early separation of camel calves is crucial in both intensive and extensive production systems. On the one hand, it is important to start early milk lactation in the intensive system or to give a chance to she-camels to recover, shorten the interval between calvings, and start the next breeding season early with a good body condition score [1]. On the other hand, in the extensive or pastoral system, it allows the she-camels to cover their nutrient requirements from the available pasture without the need for feed supplement or pasture deterioration, while conserving the feed supplement for fattening the young calves and obtaining a flavorful carcass at a young age for the market.

In the context of climate change, the significance of camels in achieving food and economic security has appeared. Camels have considerable potential as meat producers, particularly in dry regions where other livestock species are difficult to raise [2]. As a result, pastoralists all over the world, especially in Africa, are shifting their activity from cattle to camel and small ruminant production, with drought being the primary reason. Furthermore, camel calves are regarded as a cost-effective strategy for producing meat, which is essential to satisfy the increasing demand for red meat in developing countries [2, 3].

It has been reported that camels consume less feed than other ruminant species, despite their large size [4–6]. Hashi and Kamoun [7] found that camels’ dry matter (DM) intake was voluntarily limited to 1.6–1.7% of body weight (BW). This level of feed consumption is substantially less than that reported for other ruminant species [8]. Similar findings were reported for a total DM intake of less than 2% of BW for growing camels (1.64–1.93%, Shawkat et al. [9]; 1.64–1.93%, Nagpal et al. [10]; 1.8–1.2%, Emmanuel et al. [11]). Askar et al. [8] reported that a total DM intake of 2% of BW may be considered the minimal amount required for sheep or goats to meet their maintenance requirements. Camels have been noted to have lower metabolizable energy used for maintenance requirements (245–303 Kilo Joul (kJ)/kg BW^0.75^, Guerouali et al. [12]), than other ruminant species (398–450 kJ/kg BW^0.75^ for sheep and goats, Farid et al. [13]; Askar [8, 14]). In support of this point, it has been stated that camels have a lower metabolic rate than cattle and sheep [15, 16].

However, intensifying the traditional camel system [17] and employing appropriate feeding programs [18, 19] have a substantial impact on improving the growth performance and ensuring the production of heavy, high-quality, flavored carcasses for the market at a competitive price and young age. If this strategy is properly managed, camel calves can be slaughtered all year, contributing significantly to global meat self-sufficiency while taking into account the comparable meat properties of beef and camels [20, 21]. These literatures might indicate a great attention to the significance of developing camel feeding strategies for intensive management.

Therefore, the goal of the present experiment was to study the effects of feeding different levels of concentrate on growth performance and digestion of 12- to 18-month-old dromedary camel calves.

## Materials and methods

The experiment was conducted at the “National Campaign for the Promotion of Camel Productivity” farm, Ras-Sudr Research Station, which belongs to Desert Research Center, Egypt. It is about 200 km from Cairo, Egypt’s capital, at coordinates 29, 35, 30 N and 32, 42, 20 E, on the western coastal road to South Sinai Governorate. It is called a desert climate with almost complete absence of precipitation throughout the year.

### Animals and treatments

Eighteen one-year-old male camel calves, with an average BW of 298.0 ± 8.73 kg, were used in the current experiment to study the effects of feeding different levels of concentrate supplement on growth performance and digestion at different ages. Animals were divided into three treatments, six per each. Animals in each group were housed in a half-shaded, sandy floor pen (8*8 m²) for the duration of the experiment. The concentrate was administrated based on their BW at 0.7 (low), 1.0 (medium), and 1.3% (high) with free access to alfalfa hay and water. Animals individually received the concentrate supplement, as per treatment, twice a day at 08:00 and 14:00 h. The proximate analysis of concentrate and alfalfa hay is presented in Table 1.

**Table 1:** The chemical composition of concentrate feed and alfalfa hay, based on a dry matter (DM) basis

Ingredients	*Concentrate feed	Alfalfa hay	
**Dry matter**, g/ kg fresh matter	946		938
**Gross energy**, MJ/ kg DM	17.7		14.3
**Chemical composition**, g/ kg DM			
Organic matter	874		809
Crude protein	156		141
Neutral detergent fiber	342		464
Acid detergent fiber	125		252
Acid detergent lignin	37.1		59.1

*The concentrate consisted of 55% corn, 15% soybean meal, 10% cottonseed meal, 15% wheat bran, 2.5% limestone, 1.5% salt, 0.5% sodium bicarbonate, 0.1% yeast, 0.1% antitoxins, and 0.3% premix

### Experimental procedures

The study lasted six months from April to October 2019 in which the camel calves were weighed weekly and the corresponding concentrate was directly calculated and applied. The digestibility trials were conducted at two, four, and six months of the experimental period, which corresponds to camel calves aged 14, 16, and 18 months, to explore the effects of concentrate level and animal age on nutrient digestibility. Five calves in each treatment were then fitted with fecal bags and allowed to adjust to the new setting before fecal matter collection began for the following seven-day measurement period. Animals were weighed at the start and end of each trial. The amount of feed offered and refused was recorded regularly on a daily basis.

Throughout the collecting period, fecal bags were emptied twice daily, at 06:00 and 18:00, and the total amount of feces was recorded. A subset (10%) of each camel calf was taken to form a unique composite sample. Feed and feces samples were air-dried at 65◦C for 48 h and stored for subsequent analysis. The digestibility was estimated using the acid-insoluble ash corrected for its fecal recovery [18].

### Weather data

Daily ambient temperature (T◦C) and relative humidity (RH%) [22] were employed to compute the temperature–humidity index (THI) [23].

### Rumen and blood metabolism

At the end of the experiment, the rumen liquor and blood samples were taken 2–3 h after the morning feeding. Blood samples were obtained via jugular vein puncture using heparin as the anticoagulant and centrifuged at 1046 × g for 20 min. The plasma was separated and samples stored at − 20◦C for the subsequent spectrophotometric measurements of the total protein (CAT. No. TP 2020; [24]), urea (CAT. No. UR 2110; [25]), and creatinine (CAT. No. CR 1251; [26]) contents using the Biodiagnostic laboratory kits. Moreover, the rumen liquor samples were withdrawn via a stomach tube and filtered through two cheesecloth layers. A three-quarter-inch tube, one and a half meters long, was used to get the rumen samples. The animal was seated, and the tube was slowly inserted from the mouth, through the neck, and into the rumen, until the smell of methane appeared. The animal’s neck was tilted forward, and the tube was then closed and withdrawn with enough amount of rumen fluid. A digital pH meter (WPA CD70) was used to record the rumen liquor pH. Two samples, 10 and 4 mL, of rumen fluid were taken out and added to 10 mL of 0.1 N HCl, 1 mL of 2 g/L mercuric chloride, and 20 mL/L orthophosphoric acid solution. The mixture was then frozen for later analyses.

### Analytical procedures

Feeds, orts, and feces samples were analyzed for DM, organic matter (OM), gross energy (GE), crude protein (CP) [27], neutral detergent fiber (NDF) [28], and acid detergent fiber (ADF) [27], using the filter bag, ANKOM Technology Corp., USA, and acid detergent lignin (ADL) [29]. Rumen samples were centrifuged at 13,000 rpm to determine rumen ammonia [27] and volatile fatty acids [30]concentrations.

### Statistical analysis

The MIXED procedure [31] was utilized for statistical analysis of the digestibility trial data. Feeding treatments and animal age were the fixed factors, while age was a repeated measure and animal was the random effect. The model is Yijk = µ + Ti + Aj + (TA)ij + eijk, in which Yijk was the dependent variable, µ the overall mean, Ti the treatment effect, Aj the age effect, (TA)ij the interaction effect between Ti and Aj, and eijk is the residual. A one-way ANOVA was used to statistically analyze the data on growth performance, rumen fermentation, and blood metabolites, with feeding treatment acting as a fixed effect. The least significant difference with a protected F-test was used to determine differences between means.

## Results

### Climate conditions

**Fig. 1:**
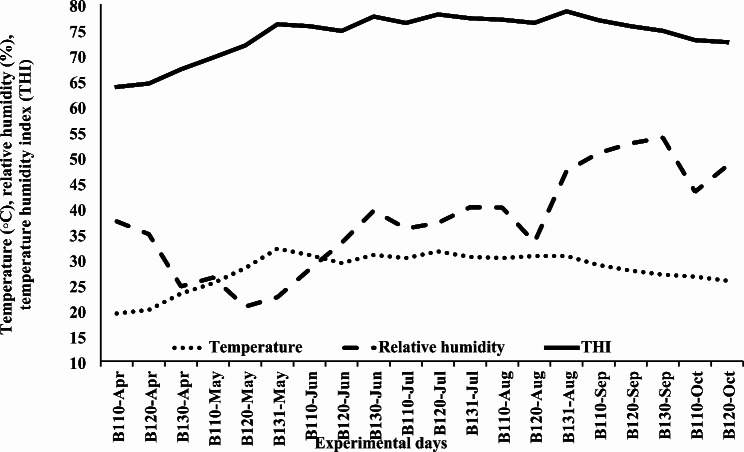
Mean temperature, relative humidity, and temperature–humidity index (THI) in 10-day periods throughout the experimental period that growing camel calves were exposed to April–October

The daily means of T, RH, and THI were determined. The average T was 27.9 °C, while the RH was 37.5% and the THI was 73.8% (Fig. 1).

### Growth performance

**Fig. 2:**
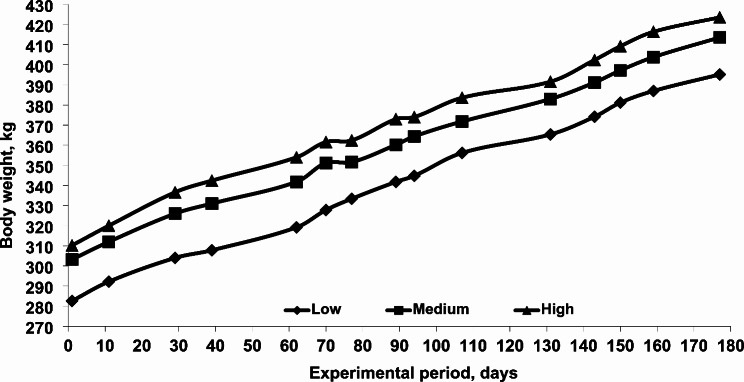
Effect of feeding different concentrate levels on body weight changes of growing camel calves

The growth performance of the 12- to 18-month-old camel calves over the 6-month experimental period is shown in Table 2. The data revealed that initial BW did not differ significantly among feeding treatments. Although animals received different levels of concentrate in their diets, there were no significant differences in final BW or BW changes (Fig. 2), resulting in a similar daily gain.

Increasing the concentrate level had a detrimental impact on forage intake that was greater (*P* < 0.05) for low vs. medium or high concentrate levels, which had comparable values. However, total intake was significantly (*P* < 0.05) higher by increasing the concentrate level, which resulted in a worse (*P* < 0.05) feed conversion ratio (FCR) for those fed a medium or high concentrate level.

**Table 2:** Growth performance of growing camel calves fed different concentrate levels in the diet along with ad lib alfalfa hay

Items	Concentrate levels (% of body weight)			SEM	*P*-value	
	Low (0.7)	Medium (1.0)	High (1.3)			
**Fattening period**,** days**	176	176	176			
**Initial body weight**,** kg**	280.6	303.2	310.2	15.05	*P* > 0.05	
**Final body weight**,** kg**	391.2	414.6	422.6	19.45	*P* > 0.05	
**Body weight changes**,** kg**	110.6	111.4	112.4	4.92	*P* > 0.05	
**Average daily gain**,** g/ day**	628.4	632.9	638.6	27.94	*P* > 0.05	
**Dry matter intake**,** kg/ day**						
Concentrate	2.166^b^	3.304^a^	3.632^a^	0.147	*P* < 0.05	
Roughage	3.364^a^	2.632^c^	3.010^b^	0.093	*P* < 0.05	
Total	5.530^b^	5.936^b^	6.642^a^	0.198	*P* < 0.05	
**Dry matter intake**,** % BW**						
Concentrate	0.65^c^	0.92^ab^	1.00^a^	0.027	*P* < 0.05	
Roughage	1.01^a^	0.74^b^	0.82^b^	0.037	*P* < 0.05	
Total	1.66^b^	1.66^b^	1.82^a^	0.051	*P* < 0.05	
**Dry matter intake**,** g/ kg BW**^**0.75**^						
Concentrate	27.7^c^	40.0^b^	43.5^a^	1.08	*P* < 0.05	
Roughage	43.0^a^	32.2^bc^	36.0^b^	1.28	*P* < 0.05	
Total	70.7^b^	72.2^b^	79.5^a^	1.65	*P* < 0.05	
**Feed conversion ratio**						
Total feed intake/ gain	8.82^c^	9.38^b^	10.42^a^	0.235	*P* < 0.05	
Roughage intake/ gain	5.37^a^	4.21^b^	4.72^b^	0.294	*P* < 0.05	
Concentrate intake/ gain	3.45^c^	5.22^b^	5.70^a^	0.132	*P* < 0.05	
**Concentrate/ roughage ratio**						
Concentrate, %	39.2^b^	55.5^a^	54.7^a^	1.21	*P* < 0.05	
Roughage, %	60.8^a^	44.5^b^	45.3b	1.21	*P* < 0.05	

^a, b,c^ Means having different superscripts within the same row differed significantly (*P*<0.05). SEM = Standard error of means

### Digestibility trials

#### Effects of concentrate level

The effects of animal age and concentrate level on intake and nutritional digestibility are summarized in Table 3.1 and 3.1. In general, no interaction between concentrate level and animal age was detected. The concentrate level had a great detrimental effect on forage intake (g/d or g/kg BW^0.75^, *P* < 0.05). This reduction in forage intake significantly impacted the roughage/concentrate ratio (*P* < 0.05), which was associated with a significant reduction in digestibility (*P* < 0.05), particularly fiber fraction digestibility. Owing to the significant refusals of concentrate with the high-concentrate camel calves, similar intakes of concentrate or forage were recorded (g/d or g/kg BW^0.75^) between those receiving medium or high concentrate levels.

**Table 3.1:** Feed intake and apparent digestibility of growing camel calves fed different concentrate levels in the diet, along with ad lib alfalfa hay at different ages (the main effects)

Item	Animal age (Months)			SEM	Concentrate levels (% of body weight)			SEM	*P*-value		
	14	16	18		Low (0.7)	Medium (1.0)	High (1.3)		Age	Treat.	Interaction
Dry matter intake, g/ day											
Concentrate	2991	3098	3200	174.4	2387^b^	3292^a^	3609^a^	174.4	*P* > 0.05	*P* < 0.05	*P* > 0.05
Roughage	3965^a^	2893^b^	2646^b^	175.8	3674^a^	2764^b^	3065^b^	175.8	*P* < 0.05	*P* < 0.05	*P* > 0.05
Total	6955^a^	5990^b^	5845^b^	268.7	6061	6056	6674	268.7	*P* < 0.05	*P* > 0.05	*P* > 0.05
**Organic matter intake**,** g/ day**	6258^a^	5385^b^	5306^b^	342.1	5471	5460	6019	342.1	*P* < 0.05	*P* > 0.05	*P* > 0.05
**Crude protein intake**,** g/ day**	990^a^	865^b^	848^b^	54.8	857	879	968	54.8	*P* < 0.05	*P* > 0.05	*P* > 0.05
**Neutral detergent fiber**,** g/ day**	2963^a^	2507^b^	2431^b^	160.3	2601	2521	2779	160.3	*P* < 0.05	*P* > 0.05	*P* > 0.05
**Dry matter intake**,** g/ kg BW**^**0.75**^											
Concentrate	37.3	36.9	36.4	1.82	29.8^b^	39.0^a^	41.9^a^	1.82	*P* > 0.05	*P* < 0.05	*P* > 0.05
Roughage	50.0^a^	34.9^b^	30.4^b^	2.14	46.6^a^	33.1^b^	35.6^b^	2.14	*P* < 0.05	*P* < 0.05	*P* > 0.05
Total	87.3^a^	71.9^b^	66.8^b^	2.88	76.3	72.1	77.5	2.88	*P* < 0.05	*P* > 0.05	*P* > 0.05
**Concentrate/ roughage ratio**											
Concentrate, %	43.1^b^	51.8^a^	54.6^a^	1.71	40.0^b^	54.9^a^	54.6^a^	1.71	*P* < 0.05	*P* < 0.05	*P* > 0.05
Roughage, %	56.9^a^	48.1^b^	45.3^b^	1.73	59.9^a^	45.0^b^	45.4^b^	1.73	*P* < 0.05	*P* < 0.05	*P* > 0.05
**Apparent nutrient digestibility**,** g/kg**											
Dry matter	695	706	708	0.48	715^a^	704^a^	689^b^	0.48	*P* > 0.05	*P* < 0.05	*P* > 0.05
Organic matter	737	730	738	0.52	744^a^	740^a^	721^b^	0.52	*P* > 0.05	*P* < 0.05	*P* > 0.05
Energy	70.9	71.3	72.4	0.76	72.6^a^	71.7^ab^	70.4^b^	0.76	*P* > 0.05	*P* < 0.05	*P* > 0.05
Crude protein	682	710	719	1.82	713	709	689	1.82	*P* > 0.05	*P* > 0.05	*P* > 0.05
Neutral detergent fiber	596^a^	539^b^	540^b^	0.75	589^a^	562^b^	524^c^	0.75	*P* < 0.05	*P* < 0.05	*P* > 0.05

^a, b, c^ Means without a common superscript letter in the row are differed (*P* < 0.05) between camel age or treatments. SEM = Standard error of means

**Table 3.2:** Feed intake and apparent digestibility of growing camel calves fed different concentrate levels in the diet, along with ad lib alfalfa hay at different ages (the interaction)

	Age, (Months)	Concentrate level (% of body weight)			SEM	*P*-value		
		Low (0.7)	Medium (1.0)	High (1.3)		Age	Treat	Interaction
Dry matter intake, g/ day								
Concentrate	14	2206	3301	3464	302.1	*P* > 0.05	*P* < 0.05	*P* > 0.05
	16	2334	3365	3594				
	18	2620	3210	3768				
Roughage	14	4467	3458	3961	304.5	*P* < 0.05	*P* < 0.05	*P* > 0.05
	16	3524	2480	2675				
	18	3023	2355	2559				
Total	14	6673	6759	7425	465.3	*P* < 0.05	*P* > 0.05	*P* > 0.05
	16	5858	5845	6268				
	18	5643	5565	6327				
**Organic matter intake**,** g/ day**								
	14	6019	6078	6678	592.5	*P* < 0.05	*P* > 0.05	*P* > 0.05
	16	5273	5250	5632				
	18	5119	5053	5747				
**Crude protein intake**,** g/ day**								
	14	935	971	1063	95.0	*P* < 0.05	*P* > 0.05	*P* > 0.05
	16	829	852	914				
	18	808	812	926				
**Neutral detergent fiber**,** g/ day**								
	14	2904	2895	3140	277.7	*P* < 0.05	*P* > 0.05	*P* > 0.05
	16	2512	2417	2593				
	18	2387	2300	2605				
**Dry matter intake**,** g/ kg BW**^**0.75**^								
Concentrate	14	28.9	40.9	42.1	3.15	*P* > 0.05	*P* < 0.05	*P* > 0.05
	16	29.2	40.0	41.4				
	18	31.0	36.1	42.0				
Roughage	14	59.3	42.8	47.6	3.70	*P* < 0.05	*P* < 0.05	*P* > 0.05
	16	44.3	29.6	30.8				
	18	36.0	26.8	28.3				
Total	14	88.3	83.7	89.7	4.9	*P* < 0.05	*P* > 0.05	*P* > 0.05
	16	73.6	69.6	72.2				
	18	67.0	62.9	70.4				
**Concentrate/ roughage ratio**								
Concentrate, %	14	33.4	49.3	46.5	2.96	*P* < 0.05	*P* < 0.05	*P* > 0.05
	16	40.1	57.7	57.7				
	18	46.4	57.8	59.7				
Roughage, %	14	66.4	50.6	53.6	3.00	*P* < 0.05	*P* < 0.05	*P* > 0.05
	16	59.8	42.2	42.4				
	18	53.4	42.2	40.2				
**Apparent nutrient digestibility**,** g/kg**								
Dry matter	14	711	697	675	0.83	*P* > 0.05	*P* < 0.05	*P* > 0.05
	16	719	707	691				
	18	714	709	699				
Organic matter	14	752	744	713	0.89	*P* > 0.05	*P* < 0.05	*P* > 0.05
	16	739	734	716				
	18	740	741	732				
Energy	14	728	706	694	9.3	*P* > 0.05	*P* < 0.05	*P* > 0.05
	16	723	717	699				
	18	727	728	718				
Crude protein	14	699	689	658	3.14	*P* > 0.05	*P* > 0.05	*P* > 0.05
	16	746	723	660				
	18	694	712	749				
Neutral detergent fiber	14	641	588	558	1.29	*P* < 0.05	*P* < 0.05	*P* > 0.05
	16	567	547	499				
	18	557	549	513				

SEM = Standard error of means

#### Effects of animal age

The intake of concentrate (g/d or g/ kg BW^0.75^) was not altered by animal age, while forage intake (g/ d or g/ kg BW^0.75^) was negatively influenced (*P* < 0.05) with advanced age. This significant decline in forage intake was accompanied by a significant decline in total intake (*P* < 0.05) and the dietary roughage-to-concentrate ratio (*P* < 0.05), which significantly impacted the fiber digestibility (*P* < 0.05). However, animal age had no detrimental impact on DM, OM, or CP digestibility (Table 3.1).

### Rumen and blood metabolites

Table 4 shows the effects of concentrate level on the blood and rumen metabolites of growing camels calves. Increasing the concentrate level significantly increased plasma protein (*P* < 0.05) and urea (*P* < 0.05) concentrations but did not affect creatinine concentration (Table 4). Rumen ammonia concentrations followed a similar trend as plasma protein and urea (*P* < 0.05, Table 4). However, a greater pH (*P* < 0.05) was found in camel calves fed low vs. medium or high levels of concentrate, which was related to a decrease (*P* < 0.10) in ruminal volatile fatty acids concentration. These changes in blood and rumen metabolism corresponded closely to variations in feed consumption attributed to concentrate addition.

**Table 4:** Blood and rumen metabolites of growing camel calves fed different concentrate levels in the diet, along with ad lib alfalfa hay

Item	Concentrate level (% of body weight)			SEM	*P*-value
	Low (0.7)	Medium (1.0)	High (1.3)		
Blood parameters					
Total protein, g/ dl	5.34^b^	5.78^ab^	6.14^a^	0.272	*P* < 0.05
Urea, mg/ dl	38.5^b^	44.2^a^	45.4^a^	1.74	*P* < 0.05
Blood urea nitrogen, mg/ dl	18.0^b^	20.7^a^	21.2^a^	0.80	*P* < 0.05
Creatinine, mg/ dl	1.60	1.60	1.62	0.114	*P* > 0.05
**Rumen parameters**					
pH	6.24^a^	5.54^b^	5.34^b^	0.11	*P* < 0.05
Ammonia, mg/dl	36.9^c^	42.3^b^	45.0^a^	0.84	*P* < 0.05
Volatile fatty acids, m.eq/dl	19.8^b^	25.2^ab^	28.0^a^	2.81	*P* > 0.05

^a, b, c^ Means without a common superscript letter in the row are differed (*P* < 0.05). SEM = Standard error of means

## Discussion

Although camel calves were received different levels of concentrate, similar BW changes (111.5 ± 4.92 kg), average daily gain (630.3 ± 27.9 g/day), and final BW were observed (Table 2). This suggests that increasing the dietary concentrate-to-roughage ratio by more than 40% has no significant effect on the performance of one-year-old camel calves (Table 2), which would negatively affect the economic efficiency of the production system. Moreover, increasing the concentrate level increased total DM intake, resulting in a worse (*P* < 0.05) FCR, 10.4 instead of 8.8 feed: gain (Table 2), which was accompanied by a decline in DM and OM digestibility (*P* < 0.05, Table 3.1) for camel calves fed a high vs. low level of concentrate respectively. When the FCR was calculated based on concentrate intake, it was 3.45 vs. 5.22 and 5.70 kg concentrate feed per kg BW gain for those fed low vs. medium or high concentrate levels respectively, which was reflected in an 18.4 or 20.5% higher cost to get one kg live BW when medium or high concentrate level was used instead of low. Similar FCR (feed: gain) were observed in growing camels at similar ages (10.0, El-Badawi and Yacout [32]; 9.6–10.1, Mohamed [33]; Shawket et al. [34]; 9.4–12.9, Emmanuel et al. [11]; 9.9–9.4, Faraz et al. [35]; 7.8, El-Badawi et al. [21]). Other research, however, have documented a higher FCR ranging from 13.3 to 16.8 for growing calves of comparable age [10, 36].

Growth performance results (Table 2) revealed that camel calves of 12–18 months old can cover their nutrient requirements for maintenance and growth when concentrate is limited to 0.7% of BW and the total DM intake is only around 1.65% of BW or 70.6 g/kg BW^0.75^ (Table 2). The current results (Table 2) are in line with the findings of Shawket et al. [9], who reported that a total DM intake of 1.64 and 1.93% of BW for growing camels at a similar age of 12 months could cover the nutrient requirements of a daily BW gain of 589 and 719 g/day, with a FCR of 8.9 and 9.1 feed: gain, respectively. Furthermore, Negpal et al. [10] found that growing camel calves at a comparable age of 15 months old, receiving a total intake of 1.38–1.55% of BW, could meet their nutrient requirements for a daily weight gain of 349–392 g/ day, with a FCR of 15.4–16.8 feed: gain. Emmanuel et al. [11] noted similar findings in 18-month-old camel calves, with a total DM intake of 1.8–2.0% of BW, a daily gain of 500–740 g, and a FCR of 9.40–12.88 feed: gain. Generally, the growth performance of camel calves is mainly influenced by sex, age, genetic potential/breed, feeding regime, and the health status of the animals [17].

Despite their large size, camels have been shown to consume less feed than other ruminants [4, 5, 18]. Similar to the current findings, Hashi and Kamoun [17] found that camels’ DM intake was voluntary limited to 1.6–1.7% of BW, which was confirmed by Gauthier-Pilters [37], Gihad et al. [38], and Askar et al. [39]. This rate of feed consumption is significantly lower than that recorded for other ruminants [6]. According to Askar [8] and Askar et al. [40], a total DM consumption of 2% of BW may be the minimal amount required for small ruminants to meet the maintenance nutrient requirements. Moreover, camels are known to have lower nutrient requirements than other ruminant. The camel’s metabolizable energy used for maintenance (MEm) is around 30–40% lower than that of small ruminants (245–303 kJ/ kg BW^0.75^ for camels, Guerouali et al. [38]; Askar et al. [41] vs. 398–450 kJ/kg BW^0.75^ for small ruminants, Farid et al. [13], 1983; Askar [8, 14]; Askar et al. [40]). In support of this, camels have been demonstrated to have lower metabolic rate than sheep and cows [15, 16]. Furthermore, camels can utilize a considerable proportion of endogenous urea, thanks to a great recycling capacity, particularly when dietary protein content is limited [42]. They may deliver urea to the gastrointestinal tract as a non-protein nitrogen source instead of excreting it as urine [43]. In general, camels seem to be more sensitive to energy deficit than protein deprivation, as they need less protein for maintenance than other ruminants [44]. In support, Nagpal [45] reported that growing camel calves, 2.5-year-old, fed a 50:50 roughage-to-concentrate ratio in a diet containing 9.9% CP at 1.43% BW could achieve a daily weight gain of 687 g with a FCR of 10.8 feed: gain. He also found that increasing the dietary protein from 9.9 to 15.4% did not improve the camel’s performance nor the digestibility in growing camel calves. Yacout and El-Badawi [46] reported that feeding camel calves (2 years old) a concentrate mixture containing 10% CP is sufficient for achieving an average daily gain of 829 g while consuming just 1.44% of BW as DM. Nagpal and Singh [47] observed similar results on 3.5-year-old growing camel calves fed complete pelleted diets at 1.47% of BW, with CP levels ranging between 8.34 and 10.40%. Furthermore, Nagpal et al. [10] found comparable results with 15-month-old growing camel fed isocaloric feed blocks containing 9.5, 12.0, and 14.5% CP at a total DM consumption of 1.47% of BW.

However, the lower total intake recorded for camel calves fed a low-concentrate (1.65% of BW or 70.6 g/kg BW^0.75^, Table 2), in comparison with those fed a high-concentrate level (1.82% of BW or 79.5 g/kg BW^0.75^), was restricted voluntarily by forage intake that was 1.01% of BW or 43.0 g/kg BW^0.75^. Roughage consumption is frequently regulated by the reticulorumen capacity and the rate of disappearance [48, 49]. Therefore, the physical fill capacity of the reticulorumen and a possible decrease in disappearance rate most likely limited the consumption of camel calves fed the high roughage diet (low-concentrate diet). The findings are comparable with those published by [7, 39], who reported that roughage consumption limits feed intake capacity in camels when the concentrate is restricted. Farid et al. [50] found that total feed intake capacity with two-year-old camel calves fed ad lib in a cafeteria feeding system was limited voluntary to 73 g/kg BW^0.75^ by roughage consumption that was varied based on its quality and type (33–45 g/kg BW^0.75^) when the concentrate feed was restricted to 75 or 50% of the ad lib feeding level. In contrast, consistent with Askar et al. [18], a medium or high concentrate level enhanced total DM intake by replacing roughage DM with concentrate (Table 2). Askar et al. [51] and Chemman et al. [52], with small ruminants, and Farid et al. [50], with camel calves, suggested that the effect of the concentrate intake level on forage consumption vary, depending on the type and quality of forage.

In this study, increasing concentrate level had a negative impact on forage consumption. A significant decrease in forage consumption was observed in growing camels receiving a medium or high concentrate level (Table 2). This resulted in a considerable drop in a roughage-to-concentrate ratio for camels fed a low vs. medium or high level of the concentrate. The results were confirmed through digestibility trials (Table 3), which were also accompanied by a significant drop in the nutrient digestibility, particularly that of fiber fractions (Table 3). The results are in line with those published by Askar et al. [39] for grazing camels, Askar et al. [18] for small ruminants, and Reis and Combs [53] for dairy cattle. The increase in dietary concentrate level was expected to have a negative impact on forage consumption and digestibility [54]. This was probably attributed to alterations in rumen fermentation [55], which may have influenced the rumination and feed residues retention time [7]. In this context, the adverse effect of concentrate on forage consumption and fiber digestibility in camel calves was clearly shown (Table 3). This may be linked to its negative effect on rumen pH (Table 4), which reduced the population and activity of cellulolytic bacteria [41, 56] and protozoa in camel calves [57]. This might reduce the efficiency of microbial protein synthesis and consequently forage utilization. However, it should be noted that growing camels can maximize the utilization of nutrients as efficiently as possible when increasing the roughage proportion of their diets from 45 to 60% (Tables 2 and 3). This could lend support to the theory that camels can extract as much energy as possible from fibrous materials and digest feeds more efficiently because their forestomachs retain feed particles longer than those of true ruminants [58].

On the other hand, limited information is available on the effect of age on concentrate intake, roughage intake, or roughage-to-concentrate ratio, which affects the economy and efficiency of camels production systems. It is well known that animals of different ages have variable nutritional requirements. The current results (Table 3.1) showed that forage consumption (g/ day or g/ kg BW^0.75^) was negatively influenced (*P* < 0.05) with advanced age. The drop in forage intake was not associated with an increase in concentrate intake (g/ kg BW^0.75^), resulting in a substantial drop in total DM intake (*P* < 0.05) and dietary roughage-to-concentrate ratio (*P* < 0.05). Young animals, in general, are incapable of dealing with large amounts of roughage as effectively as older animals, which contradicts our findings. It is worth noting that camel calves at a young age prefer to satisfy their nutrient requirements from alfalfa hay rather than concentrate (Table 3.1). This could provide a significant economic advantage to growing camel production systems, as studies on the relationship between camel age, nutrient requirements, and feeding behavior have yet to be developed.

It is expected that the increase in BW from 12 to 18 months of age (Table 2) is supposed to be linked to an increase or at least a similar DM intake in relation to BW (g/ kg BW^0.75^) with advanced age. This was true; it happened with the concentrate intake but not with the roughage consumption, which negatively affected the total DM intake (Table 3).

However, the results of T, RH, and THI (Fig. 1) revealed the hottest climatic conditions, which lasted from June to September. The increase in T and THI throughout the drought season was obviously related to the heat load on animals, which might have played a great role in the significant decline in total feed intake (Table 3). The impact of heat load on intake has been clearly addressed [59]. In our research with desert animals, Helal et al. [60] found a significant relationship between energy expenditure and T and THI for desert goats but not Shami goats, implying that energy expenditure of desert goats is only sensitive to climate conditions but not the other goats. They revealed that desert Balady goats have an advantage in a reduced MEm requirement under hot temperature, whereas they have an increased MEm requirement with low T conditions. This explains the drop in feed intake shown in this experiment, which was caused by the decrease in nutrient requirements of desert camel calves under hot conditions.

However, it was expected that animals in hot weather try to dissipate excess heat in order to deal with heat stress and adapt to the hot weather conditions. Consequently, camel calves are supposed to select feed components with less energy content (alfalfa hay) instead of concentrate under hot conditions, which is also not supported by our results (Table 3.1). In this context, the concentrate was first administrated to the camel calves in the current study, followed by alfalfa hay. This suggests that camels might cover the majority of their nutrient requirements from the concentrate first and the rest from the alfalfa hay. Yacout and El-Badawi [46] demonstrated that camels preferred feeding concentrates as their first option whenever they were available. They also confirmed the significance role of concentrate feed in providing growing calves with the nutrients required to achieve the target growth. The concentrate feed is considered a good source of volatile fatty acids, which are the primary source of energy for young camels and play a significant role in promoting rumen development [61, 62, and 63], while neither the quantity [64] nor the source of forage [65] seem to play a substantial role in rumen development. Similar DM and OM digestibility observed at different ages (Table 3) suggests that rumen function was fully developed at an earlier age [66].

However, regardless of the amount of concentrate received, a significant decrease in forage consumption with advanced age had a detrimental influence on total DM consumption and the roughage-to-concentrate ratio, which was associated with a significant reduction in fiber digestibility, as expected (Table 3). As mentioned, this could be related to its detrimental impact on rumen pH, which in turn decreased the number and activity of cellulolytic bacteria and protozoa [41, 56, 57]. It may also reduce the efficiency of microbial protein synthesis, resulting in lower forage utilization. Similar results were observed by increasing the dietary concentrate level (Table 3).

The higher plasma protein and urea concentrations of growing camel-calves supplemented with medium or high concentrate levels reflected the significant (*P* < 0.05) increase in the concentrate level and consequently the protein intake (Table 3) that was not linked to the significant increase in the final BW (Table 2). The results of urea concentration followed a trend similar to that of the rumen ammonia concentrations. Increasing the concentrate consumption increased the amount of protein degradation in the rumen, which reflected in increased ammonia release. This ammonia is primarily reutilized by rumen microorganisms for microbial protein production, while the rest is converted into urea in the liver and mostly excreted in most ruminant species, except for camels. Contrary to the conventional concept, the regulatory mechanism for urea excretion in camels appears to operate independently of the plasma urea level. This is evident in the fact that, under conditions of dehydration, very low urea clearances continue, despite a steadily increasing plasma urea concentration. The only factor found to be related to the renal excretion of urea was the need for protein [43]. When the need for protein is high on account of low protein consumption or rapid growth, as in this study (Table 2), the percentage of filtered urea excreted decreases to very low values. Camels may transfer urea to the gastrointestinal tract as a non-protein nitrogen source instead of being excreted as urine [67]. They have potent urea-recycling mechanisms, up to 90% of blood urea nitrogen, compared to other ruminants, which explains the similar daily gain and final BW among feeding treatments (Table 2) that is not related to the plasma protein concentration results (Table 4).

A continuous decrease in ruminal pH was reported with increasing the level of concentrate, which was associated with an increase in the total volatile fatty acids (Table 4). This might be because of the availability of a large amount of fermentable carbohydrate when the level of concentrate is increased in the diet [68–70], particularly that of propionate-to-acetate ratio [41] in camel calves. Askar et al. [66] reported that rumen pH levels below 6 have negative effects on rumen fermentation and animal performance. This suggests that giving growing camel calves a high-concentrate level is not an appropriate option, which is consistent with the current findings related to FCR (Table 2) and digestibility of nutrients (Table 3.1).

## Conclusions

In deserts or dry regions where cattle cannot be raised, the present study concluded that yearling camel calves receiving different levels of concentrate with ad lib alfalfa hay could cover their nutrient requirements for maintenance and growth with an average daily gain of 630 g/day when the level of concentrate was limited to 0.7% of body weight and the total intake was only around 1.65% of body weight, or 70.6 g/kg metabolic body weight.

## Data Availability

Data is provided within the manuscript. All data and materials are owned by the authors and/or no permissions are required.

## Abbreviations

ADF: Acid detergent fiber
ADL: Acid detergent lignin
BW: Body weight
CP: Crude protein
DM: Dry matter
FCR: Feed conversion ratio
GE: Gross energy
kJ: Kilo Joul
Mem: Metabolizable energy used for maintenance
NDF: Neutral detergent fiber
OM: Organic matter
RH: Relative humidity
SEM: Standard error of means
T: Ambient temperature
THI: Temperature–humidity index
